# Resting time after phorbol 12-myristate 13-acetate in THP-1 derived macrophages provides a non-biased model for the study of NLRP3 inflammasome

**DOI:** 10.3389/fimmu.2022.958098

**Published:** 2022-12-22

**Authors:** Sonia Giambelluca, Matthias Ochs, Elena Lopez-Rodriguez

**Affiliations:** ^1^ Institute of Functional Anatomy, Charité - Univeristätsmedizin Berlin, Berlin, Germany; ^2^ German Center for Lung Research (DZL), Berlin, Germany

**Keywords:** phorbol 12-myristate 13-acetate (PMA), interleukin-1β (IL-1β), pro-caspase-1, cell differentiation, macrophage surface markers, monocyte - macrophage, *in vitro* model, ASC speck

## Abstract

**Background:**

The activation of NLRP3 inflammasome in macrophages has been proven to play a crucial role in the development of cardiovascular diseases. THP-1 monocytes can be differentiated to macrophages by incubation with phorbol-12-myristate 13-acetate (PMA), providing a suitable model for *in vitro* studies. However, PMA has been shown to have effects on the levels of IL-1β, the main mediator of NLRP3 inflammasome, while the effects on the other mediators of the inflammasome have not been reported before.

**Methods:**

THP-1 monocytes were incubated without (THP-1), with 5ng/ml PMA for 48h (PMA48h) or with 5ng/ml PMA for 48h plus 24h in fresh medium (PMArest). Morphological changes and the expression of macrophage surface markers (CD14, CD11b, CD36 and CD204) were evaluated by flow cytometry. Changes in intracellular levels of inflammasome components (NLRP3, ASC, pro-caspase-1, pro-IL1β) were analyzed by western blot and release of mature IL-1β in cell supernatant was analyzed by ELISA. ASC speck formation was determined by immunofluorescence.

**Results:**

After 48h incubation with PMA or subsequent rest in fresh medium, cells became adherent, and the differential expression of CD36, CD11b, CD14 and CD204 compared to THP-1 cells confirmed that PMArest resemble macrophages from a molecular point of view. Changes in the levels were detected in PMA48h group for all the NLRP3-related proteins, with increase of NLRP3 and pro-IL-1β and secretion of mature IL-1β. In PMArest, no pro-IL-1β and lower amounts of mature IL-1β were detected. No ASC speck was found in PMA treated groups, but the addition of a second stimulus to PMArest resulted in ASC speck formation, together with IL-1β production, confirming the responsiveness of the model.

**Conclusion:**

Differentiation of THP-1 with 5ng/ml PMA followed by 24h resting period provides a model that morphologically and molecularly resembles macrophages. However, even at low concentrations, PMA induces production of IL-1β. The 24h rest period provides for down-regulation of pro-IL-1β in PMArest group, without affecting its ability to respond to a second stimulus through activation of inflammasome.

## 1 Introduction

The Nod-like receptor family pyrin domain-containing 3 (NLRP3) inflammasome is a multiprotein complex that mediates caspase-1 activation, *via* apoptosis-associated speck like protein containing a caspase recruitment domain (ASC), in response to microbial infection and cellular damage. Upon assembly of the inflammasome, ASC is mobilized from his soluble cytoplasmic form to assemble into a large singular protein complex of about 1µm diameter, termed “speck”, essential for caspase-1 activation, thus playing a critical role in innate immune response (1). Indeed, activated caspase-1 is involved in the maturation of proinflammatory cytokines, such as interleukin-1β (IL-1β) and IL-18, and in the promotion of pyroptotic cell death (pyroptosis), through cleavage and activation of gasdermin D.

Activation of the NLRP3 inflammasome has been shown to contribute to the development of a number of cardiovascular diseases (CVDs), representing a key pathogenetic mechanism in the formation and progression of atherosclerosis, and in the myocardial response to ischemic and nonischemic injury (2, 3). Moreover, in the last decade the effectiveness of NLRP3 inhibitors as therapeutic treatment for CVDs has been extensively investigated (4, 5).

The growing interest in the role of NLRP3 inflammasome in the onset of CVDs has given rise to a copious amount of *in vitro* studies investigating the mechanisms underlying its activation and regulation. Among the models employed, peripheral blood mononuclear cell (PBMC) and monocyte cell lines, such as THP-1 and U937, are the most frequent (6–10). PBMC are routinely used to derive monocytes and macrophages, while THP-1 can be differentiated into macrophage-like cells by using phorbol 12-myristate 13-acetate (PMA). After exposure to PMA, THP-1 cells start to adhere to culture plates and show morphological and functional changes, including flat and amoeboid shape and increased cell surface expression of macrophage markers, resulting in a phenotype that resembles PBMC-derived macrophages (11). The advantages of using THP-1 cell line over human PBMC-derived monocytes or macrophages have been recently reviewed by Chanput et al. (12), and include: a higher growing rate compared to that of PBMC-derived macrophages, stability in cell sensitivity and activity also when cultured for long term (up to passage 25), possibility to be stored for years in liquid nitrogen, and a homogeneous genetic background, thus facilitating the reproducibility of findings. Moreover, contamination with other blood components when isolating PBMC from buffy coat should be taken into account.

Although the convenience of using THP-1 cells as a model is widely accepted, when referring to the method used for the differentiation of THP-1 monocytes into macrophages, the lack of a standardized protocol may give rise to inconsistent and non-reproducible results among different studies. The multitude of applied protocols in the literature ranges from 5 to 400ng/mL PMA for 1–5 days of incubation (13–15), without concern for possible upregulation of some genes and changes in cytokine profile (16, 17). Indeed, PMA has been proven to have dose-dependent effects on the level of several molecules, included IL-1β (18–20). When aiming to investigate the activation of NLRP3 as a response to a specific treatment or to a particular experimental condition, it appears clear that the use of PMA as a differentiation agent deserves careful considerations. High concentrations or long exposure may represent potential sources of bias, making unclear if the triggering or release of the molecules of interest are a result of the biological question or of the bias itself. In this case, as IL-1β is one of the outcomes of PMA exposure, the use of IL-1β alone as primary read out of inflammasome activation may be biased.

In this paper, we investigated the effect of exposing THP-1 to PMA at 5ng/ml for 48h, with or without a rest period of 24h without stimulus, on the main components of NLRP3 pathway. This differentiation protocol has been previously reported to be the minimum amount of PMA sufficient to induce stable differentiation without undesirable gene upregulation upon secondary stimuli (20). However, the effect on the main mediators of the NLRP3 pathway has never been reported. An optimized protocol may provide an *in vitro* macrophage model that is not only practical to use but can also better reflect the physiologic conditions with minimized risk of biased results.

## 2 Material and methods

### 2.1 Cell culture and treatment

THP-1 (ATCC^®^ TIB202™, Virginia) monocytes were cultured in RPMI-1640 complete medium with L-glutamine, 25 mM HEPES (Corning, Virginia) containing 10% FBS (Sigma Aldrich, Germany) and 1% penicillin/streptomycin (10000 units penicillin + 10mg streptomycin/ml, Sigma Aldrich, Germany), plated at a density of 2 x 10^5^ cells/ml in T25 flasks (Eppendorf, Germany), and incubated at 37°C in 5% CO_2_. Cells from passage p8 to p20 were used for the study.

THP-1 monocytes were induced to differentiate to macrophages-like cells (MLCs) by incubation of 5 x 10^5^ cells in a 6-well plate in 2ml complete medium without (THP-1) or with 5ng/ml of PMA (Stemcell technology, Switzerland) in dimethyl sulfoxide (final concentration 0.1%, DMSO, Sigma Aldrich, Germany) for 48h (PMA48h), or with 5ng/ml PMA for 48h plus 24h in fresh medium (PMArest). DMSO to a final concentration of 0.1% was added to THP-1 group to exclude differences in marker expressions due to the presence of DMSO. The chosen dose of 5ng/ml PMA has been previously reported as the minimal concentration of PMA for stable differentiation since cell adherence, a hallmark of differentiation, is unstable at lower concentration (19).

THP-1 cells differentiation to MLCs was evaluated at 48h (PMA48h) after incubation with PMA and 24h after stimulus removal (PMArest) by means of adherence test and fluorescence-activated cell sorting (FACS) analysis, as described below.

To obtain a positive control for the validation of the methods used to study the inflammasome activation, THP-1 cells were seeded in the presence of PMA 50ng/ml overnight followed by incubation with lipopolysaccharide (LPS) from Escherichia coli O26: B6 ≥10000EU/mg (Sigma Aldrich, Germany) (5µg/ml in phosphate-buffered saline, PBS) for 3h in complete media (CTLR+). The CTRL+ sample was only used for methods validation and not included in the statistical analysis.

### 2.2 Adherence test

The adherence test was performed after 48h incubation with 5ng/ml PMA in PMA48h group and after 24h rest in fresh medium for THP-1 and PMArest groups. The medium was collected in 15ml tubes and non-adherent cells were centrifuged at 200 xg for 4min at room temperature. The supernatant was collected and stored at -20°C until further ELISA analysis; non-adherent cells were resuspended in 200µl medium and counted to determine the adherence percentage. Adherent cells were detached by trypsinization with 1ml Trypsin EDTA 0.25% (Sigma Aldrich, Germany) for 10min at 37°C, collected by centrifugation, resuspended in 200µl medium, and counted. Results are expressed as percentage of total counted cells.

An automated Corning Cell Counter (CytoSMART Technologies, Netherland) was employed to calculate the number and viability of cells after trypan blue (Sigma Aldrich, Germany) staining.

Non-adherent cells, for THP-1 group, and adherent cells, for PMA48h and PMArest groups, were then pelleted again and stored at -20°C until western blot analysis.

Changes in morphology were detected by hematoxylin and eosin staining. Cells were seeded at a density of 150000 cells/ml in a 24-wells and treated as reported above. At collection time, cells were fixed in formalin 4%, washed twice in PBS, then stained as previously described (21). Images were acquired by Axio Imager.Z1 (Zeiss, Germany) and analyzed by ZEN software (v3.4, blue edition, Zeiss, Germany).

### 2.3 Fluorescence-activated cell sorting analysis

To confirm the differentiation into macrophages, morphological changes and macrophage marker expression were characterized on THP-1 (non-adherent) and PMA48h and PMArest (adherent) cells by FACS.

The combined positive staining for CD36, CD14, CD11b, and CD204 determined the differentiation into MLC (14, 20, 22, 23). Briefly, cells were collected as reported for adherence test, pelleted by centrifugation at 200 xg for 4min at room temperature, washed twice with wash buffer (5% FBS in PBS), and incubated for 30min at 4°C in wash buffer containing conjugated antibodies: anti-CD36 – PE (1:1000), anti-CD14 – APC (1:500), anti-CD11b - PE-Vio^®^ 770 (1:500), and anti-CD204 - Vio^®^ Bright FITC REAfinity (1:500) or isotype control antibody (IgG2a – PE, IgG2a – APC, IgG2b - PE-Vio^®^ 770, IgG1- Vio Bright FITC REAfinity) (Miltenyi Biotec, Germany). Following incubation, samples were centrifuged at 200 xg for 4min at room temperature and pellets were resuspended in wash buffer.

A BD FACS Canto™ II Cell Analyzer (BD Biosciences, US) was employed. For every sample, 50000 events were recorded. Cells were gated by forward scatter – area (FSC-A) vs side scatter – area (SSC-A), then by SSC-A vs side scatter-width (SSC-W) and by FSC-A vs forward scatter – width (FSC-W).

Flow cytometric data were analyzed by FlowJo™ software (v10.7.2, BD, Ashland, OR, USA).

### 2.4 Inflammasome mediators analysis

To evaluate the effect of PMA treatment on the NLRP3 inflammasome, the levels of inflammasome mediators were assessed in THP-1, PMA48h and PMArest.

Commercially available ELISA kits were used to measure the levels of secreted human IL‐1β (Human IL-1beta/IL-1F2), and caspase-1 (Human Caspase-1/ICE) (Quantikine ELISA Kit, R&D System, Minnesota, USA) in undiluted cell media, according to the manufacturer’s instructions.

Western blot analysis was used to detect NLRP3, ASC, pro-caspase-1, pro-IL-1β, and caspase 8 in cell lysate. Cell pellet was lysed by addition of 200µl RIPA lysis buffer (Thermo Scientific, Germany) containing protease inhibitor cocktail (cOmplete, Sigma Aldrich, Germany). The cell debris and nuclei were removed by centrifugation at 12000 xg for 20min at 4°C.

The protein concentration in cell lysates was determined using the Micro BCA™ Protein Assay Kit (Thermo Fisher Scientific, Germany). Proteins were denatured in 1x Laemmli buffer (Tris-HCl 63 mM, Glycerol 10%, SDS 2%, Bromophenol blue 0.01%, 2-Mercaptoethanol 5%; pH 6.8) by heating for 10min at 95°C, were then resolved by SDS-PAGE, with 8-12% (wt/vol) separating gels, and transferred to PVDF membrane. Nonspecific sites on PVDF membrane were blocked with 5% non-fat milk powder (VWR, Belgium) in TBS + 0.1% Tween for 1h at room temperature. The membranes were then incubated overnight at 4°C with primary antibodies diluted 1:1000 in blocking solution or 5% BSA in TBS + 0.1% Tween: pro-IL-1β (ab216995), NLRP3 (ab263899), and pro-caspase-1 (ab179515) (Abcam, UK), ASC/TMS1 (#13833), and caspase-8 (#9746, Cell signalling technology, Massachusetts, USA). Proteins were detected by incubation with HRP-conjugated secondary antibodies at half of the primary antibody concentration, and protein bands were visualized by ECL western blotting detection reagents (Amersham Pharmacia Biotech, UK) and enhanced chemiluminescence (ECL Chemocam imager, Intas science imaging, Germany). β-actin (ab8229, Abcam, UK) served as loading control protein (24) to normalize western blot results and was analyzed following the same protocol at a dilution of 1:1000.

### 2.5 Responsiveness test

To test if, at the end of the 24h after PMA stimulus removal, PMArest cells were able to respond to secondary stimuli and activate the inflammasome, the PMArest group was further treated with LPS at 1 or 5µg/ml for 3h alone or followed by treatment with nigericin (NIG, #66419, Cell signalling technology, Massachusetts, USA) at 5 or 10µM for 45min (25, 26). The activation of canonical inflammasome was evaluated by analysis of pro-IL-1β and released IL-1β, as described above, in PMArest treated with LPS or with LPS+NIG, compared to untreated PMArest.

### 2.6 Detection of ASC specks

Due to the large size of ASC specks, their detection can be performed by imaging-based techniques. To determine if the treatment with PMA is associated with ASC specks oligomerization, immunofluorescence was used, as previously described (26). Briefly, THP-1 were seeded at a density of 150000 cells/ml in a 24-wells plate containing a glass coverslip per well, and treated as reported above to obtain the following experimental groups: PMA48h, PMArest, PMArest treated with LPS at 1 or 5µg/ml for 3h alone or followed by treatment with NIG at 5 or 10µM for 45min. At the collection time, cells that adhered to the coverslip, were washed in PBS and fixed in PBS-buffered formalin 4% for 30min at 37°C, then blocked in blocking/permeabilization (block/perm) buffer (10% goat serum, 1% FBS, and 0.5% Triton-x100 in PBS) for 30min at 37°C. The cells were then incubated with anti-ASC/TMS1 (#13833) primary antibody at 1:1000 dilution in block/perm buffer for 1h at room temperature. The staining for ASC was performed by incubation with conjugated secondary antibody, goat-anti-rabbit-PE (#79408, Cell signalling technology, Massachusetts, USA) at 1:2000 in block/perm buffer for 1h at room temperature. Nuclei were stained by incubation with Hoechst 34580 (Chemodex, Switzerland) at 1:1000 dilution in PBS for 30min at room temperature. Images were acquired by Axio Imager.Z2 (Zeiss, Germany) and analyzed by ZEN software (v3.4, blue edition, Zeiss, Germany). Unspecific signal due to secondary antibody has been excluded by immunofluorescence analysis of the CTRL+ incubated with the secondary antibody alone in absence of primary antibody (Neg CTRL).

### 2.7 Statistical analysis

For FACS, western blot and ELISA analyses, every treatment condition was replicated in 3 different wells in the same row of the plate (technical replicates). The row represented the experimental unit. To obtain three biological replicates the experiments were repeated three times in different plates with three different cell passages and freshly prepared stimuli (N=3). Data are shown as mean of the 3 biological replicates. Descriptive statistical analysis and graphs of the results were performed by GraphPad Prism 8 Software (San Diego, CA, USA).

For the analysis of ASC specks, the images are representative of three technical replicates.

## 3 Results

### 3.1 PMArest stably differentiated into MLCs

**Figure 1:**
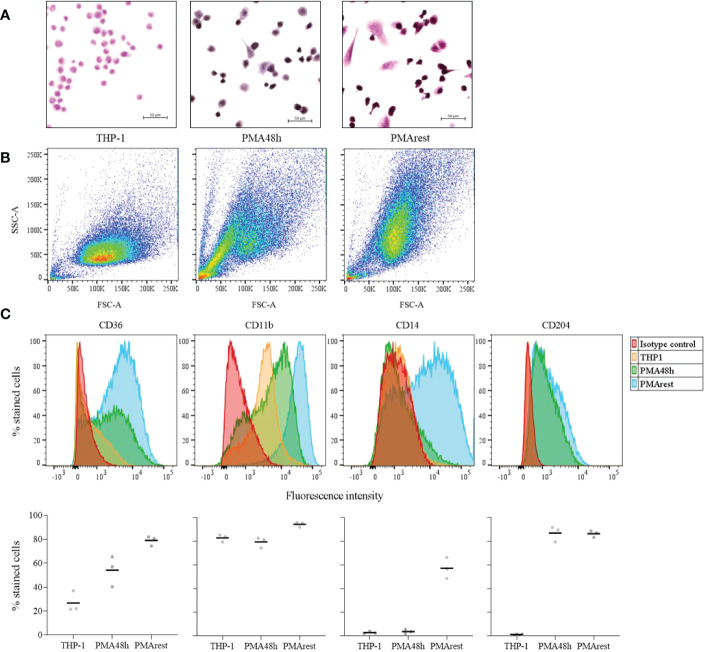
Assessment of the differentiation into macrophage-like cells. **(A)** Representative hematoxylin and eosin staining of THP-1 monocytes incubated without (THP-1), with 5ng/ml PMA for 48h (PMA48h) or with 5ng/ml PMA for 48h plus 24h in fresh medium (PMArest). All the micrographs were taken at the same magnification and reported with the same scale (scale bar = 50µm). **(B)** Representative forward (FSC-A) and side light scatter plot (SSC-A) of THP-1, PMA48h and PMArest. **(C)** Representative FACS analysis of THP-1 (yellow), PMA48h (green) and PMArest (blue) cells stained using anti- CD36, CD11b, CD14, or CD204 conjugated antibodies. For each marker, the expression was assessed by comparison with matched isotype control (red). Upper: histogram plots of marker expression (y: percentage of stained cells normalized by mode; x: fluorescence intensity); lower: mean value of the percentage of total events expressing the single markers. Data are shown as mean of three independent experiments in different plates with three different cell passages and freshly prepared stimuli (N=3).

Differentiation from monocyte to macrophage is associated with morphological changes, such as an increase in size and granularity, as well as cell adhesion, a hallmark of macrophages (11). Representative micrographs showing morphological changes associated with differentiation are reported in **Figure 1A**. After induction by 5ng/ml PMA for 48h, 98.1% of counted PMA48h cells became adherent, with a viability of 94.4%. Following 24h rest after PMA stimulus removal, 87.1% of PMArest cells were adherent and showed a viability of 97.3%, compared to 99.4% of viability in THP-1 group. Alongside adherence, the treatment with PMA resulted in cells adopting a stellate or a spindle-like morphology after 48h and subsequent rest. Moreover, the hematoxylin and eosin staining resulted in a purple staining for the PMA treated cells, compared with THP-1 assuming a lighter pink staining, indicating changes in cytoplasmic and granular composition.

Morphological changes were confirmed by FACS analysis by comparing the FSC-A and SSC-A of the 3 groups. PMA48h and PMArest showed a gradual increase in size (FSC-A) and granularity (SSC-A) compared to the THP-1 cells (**Figure 1B**). Moreover, while for PMA48h group heterogeneity in size and granularity was detected, PMArest resulted in a homogenous cell population.

The differential expression of macrophage specific surface markers was assessed by FACS. **Figure 1C** shows the histogram plots for each marker expression in THP-1, PMA48h and PMArest, assessed by comparison with matched isotype control, and the mean value of the percentage of total events expressing the single markers. THP-1 monocytes showed no surface expression of CD14, and CD204 and low surface expression of CD36, the latter being higher expressed in both PMA48h and in PMArest. In contrast to THP-1, both PMA48h and PMArest exhibited surface expression of CD204, while surface expression of CD14 was only detected in PMArest. All the treatment groups showed expression of CD11b, with enhanced intensity in PMArest.

### 3.2 IL-1β level dropped down in PMArest

**Figure 2:**
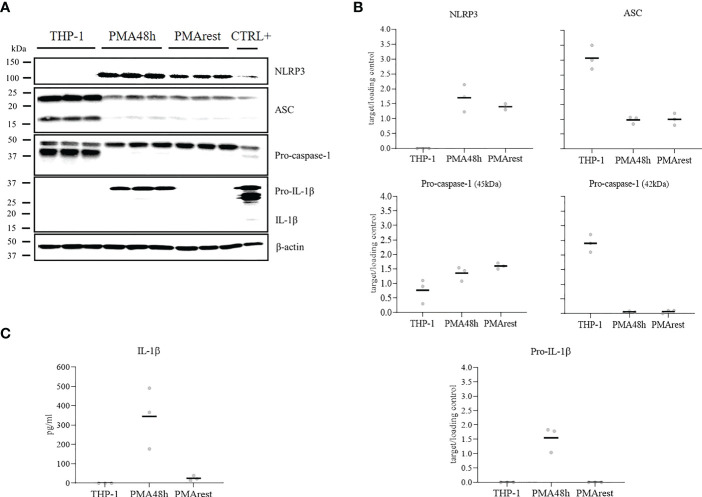
NLRP3 inflammasome mediators analysis. **(A)** Representative results of western blot analyses for NLRP3, ASC, pro-caspase-1, pro-IL-1β, and β-actin in cell lysate of THP-1 monocytes incubated without (THP-1), with 5ng/ml PMA for 48h (PMA48h) or with 5ng/ml PMA for 48h plus 24h in fresh medium (PMArest). The positive control (CTLR+) for the validation of western blot was obtained by incubation of THP-1 cells with PMA 50ng/ml overnight followed by incubation with 5µg/ml LPS for 3h in complete media. **(B)** Relative ratio of the expression of the target proteins to the loading control (β-actin), detected by western blot analysis in cell lysate of THP-1, PMA48h, and PMArest cells. **(C)** ELISA analysis of IL-1β in supernatant of THP-1, PMA48h, and PMArest cells. Data are shown as mean of three independent experiments in different plates with three different cell passages and freshly prepared stimuli (N=3).

To determine if NLRP3 inflammasome activation occurred as a consequence of PMA treatment, we compared the detection of NLRP3, ASC, pro-caspase-1, and pro-IL-1β in cell lysate and release of mature IL-1β in the supernatant of THP-1, PMA48h and PMArest. Representative results for western blot analyses of cell lysate and the relative ratio of the detected proteins to the loading control are shown in **Figures 2A, B**, respectively. Western blot analysis confirmed that levels of pro-IL-1β (predicted band size 31kDa) and NLRP3 (predicted bands size 118kDa) were undetectable in THP-1 cells, while treatment with PMA stimulated the production of both pro-IL-1β and NLRP3 in PMA48h group. Along with the enhanced production of NLRP3, a different expression pattern was detected for ASC and for the zymogen forms of caspase-1 (predicted band size 45-42kDa) in PMA48h group compared to THP-1, with 3 times lower relative amount of ASC and 2.5 times lower amount of the 42 kDa band of pro-caspase-1 in PMA48h. On the other side, a higher relative expression of the 45kDa band of pro-caspase-1 was detected in PMA48h compared to THP-1 cells. However, no mature form of caspase-1 (predicted band size 10-12kDa) was detected by western blot in the cell lysate of any of the groups.

In PMArest group, after 24h resting period without PMA, the level of pro-IL-1β became undetectable again, and a decrease in NLRP3 level compared to PMA48h was detected. In contrast, the levels of ASC and pro-caspase-1 remained comparable to the PMA48h group.

In accordance with western blot analysis, secreted IL-1β was not detectable in the supernatant of THP-1, while released IL-1β was found in the supernatant of PMA48h. Levels of IL-1β dropped in PMArest group compared to PMA48h (**Figure 2C**). No released active caspase-1 was detected by ELISA in the supernatant of any of the groups (data not shown). The validity of the ELISA assays was confirmed by analysis of IL-1β and caspase-1 in the supernatant of the CTRL+, in which a concentration of 308 ± 27pg/mL and of 328pg/mL, respectively, was measured.

### 3.3 PMArest were responsive to canonical activation of inflammasome

**Figure 3:**
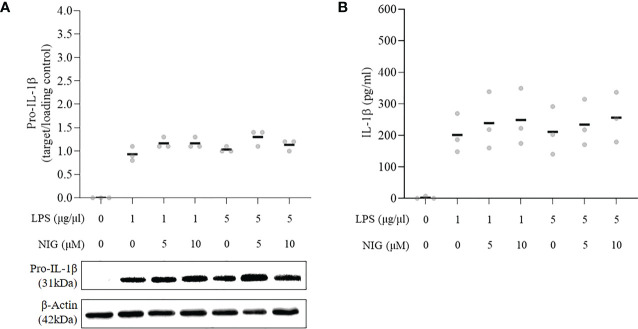
Responsiveness of THP-1 monocytes incubated with 5ng/ml PMA for 48h plus 24h in fresh medium (PMArest) to canonical inflammasome stimulus. **(A)** Relative ratio of the expression of pro-IL-1β to the loading control (β-actin), detected by western blot analysis in cell lysate and **(B)** ELISA analysis of IL-1β in supernatant of PMArest untreated or treated with LPS at 1 or 5µg/ml for 3h alone or followed by NIG at 5 or 10µM). Data are shown as mean of three independent experiments in different plates with three different cell passages and freshly prepared stimuli (N=3).

To test the ability of the PMArest cells to respond to a canonical inflammasome activation stimulus, cells were treated with LPS at 1 or 5µg/ml alone or followed by NIG at 5 or 10µM. The results of the responsiveness test are reported in **Figure 3**. At any of the conditions tested, after treatment, PMArest were able to produce and release IL-1β, as confirmed by the presence of pro-IL-1β in the cell lysate (**Figure 3A**) and the higher levels of the cytokine in their supernatant (**Figure 3B**), compared to untreated PMArest. Although a little increasing trend was found in the groups treated with NIG compared to groups treated with LPS alone, not a clear dose dependent response was found.

### 3.4 ASC specks were not immunodetected in PMArest

**Figure 4:**
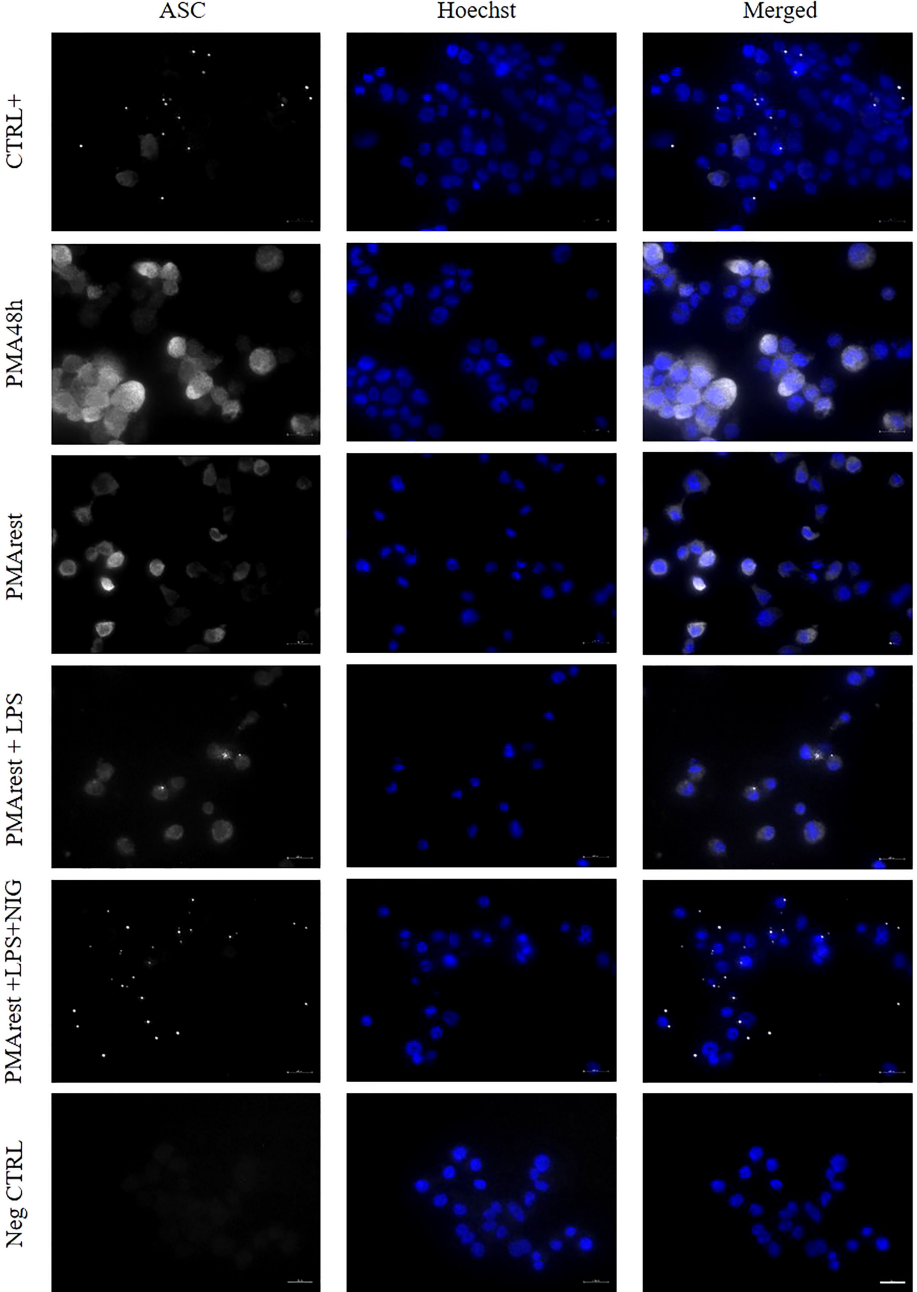
Immunofluorescence analysis of ASC specks. Representative results of ASC speck formation in (from top to bottom) THP-1 cells incubated with PMA 50ng/ml overnight followed by incubation with 5µg/ml LPS for 3h (CTRL+), 5ng/ml PMA for 48h (PMA48h), 5ng/ml PMA for 48h plus 24h in fresh medium (PMArest), PMArest treated with 5µg/ml LPS for 3h (PMArest+LPS), and PMArest treated with 5µg/ml LPS for 3h + 45min with 10µM nigericin (PMArest+LPS+NIG). Cells were fixed and stained with anti-ASC antibody, followed by staining with PE-coniugated secondary antibody (false colored in white). Nuclei were stained by incubation with Hoechst 34580 (colored in blue). As a negative control (Neg CTRL), cells were incubated with the secondary antibodies alone. All the micrographs were taken at the same magnification and reported with the same scale (scale bar = 20µm).

Oligomerization of ASC into specks, as a readout for inflammasome activation (1), was investigated in PMA48h, PMArest, PMArest treated with LPS at 1 or 5µg/ml alone or followed by NIG at 5 or 10µM. Representative results are shown in **Figure 4**. Since no appreciable differences were found among different concentrations of LPS and NIG, only the results relative to LPS 5µg/ml alone (PMArest+LPS) or followed by NIG at 10µM (PMArest+LPS+NIG) are reported in **Figure 4** and discussed. The remaining results are shown in **Supplementary Figure 3**. The staining for ASC is represented in white and nuclear staining (Hoechst) in blue. Activation of the inflammasome is characterized by a change in ASC status from diffuse cytoplasmic form to a speck, visualized as a singular perinuclear structure. The method was validated on our CTRL+ control in which a low amount of cytoplasmic ASC was detected along with several speck-like perinuclear bright dots. In PMA48h and PMArest groups, no speck structures were detected, and ASC showed a broad, weak cytosolic distribution. After treatment PMArest cells with LPS alone, showed an amount of diffuse ASC decreased compared to untreated PMArest and few specks were detected. In PMArest treated with LPS+NIG, cytoplasmic ASC was barely detectable, and instead they showed a high number of specks.

## 4 Discussion

The study aimed to assess the validity of THP-1 derived macrophages as a model to study the activation of the NLRP3 inflammasome. Several models are employed to study the inflammatory response of monocytes and macrophages, included primary PBMCs and monocyte cell lines. Due to donor variability and technical issues in long term handling of primary cells *in vitro*, THP-1 has been widely accepted as a macrophage model after differentiation by PMA treatment. However, the conditions used for differentiation, particularly the concentration of PMA and the duration of treatment, vary widely in the published literature, though a dose-dependent effect of PMA on the expression of inflammatory cytokines was already proved (20, 27). Indeed, PMA is a structural analogue of diacylglycerol, a potent activator of protein kinase-C (PKC) (28). PKC enzyme family comprise 10 cytosolic isoforms that upon activation are translocated to the plasma membrane, where they play a critical role in different signal transduction pathways and in the regulation of cell growth and differentiation. In particular, the activation of certain isoforms of PKC by PMA results in the induction of pro-inflammatory cytokines synthesis, among which IL-6, TNF-*α*, and IL-1β (29).

In our model, the differentiation of THP-1 into MLCs was obtained by treatment with 5ng/ml PMA for 48h, followed by a rest period of 24h without PMA stimulus (PMArest). The chosen dose was reported to be the minimal concentration of PMA for stable differentiation (20). Moreover, since PMA has dose-dependent effects on monocytes, it is better to use lower dosages with longer incubation time (30), thus a 48h incubation time was chosen. The differentiation into MLCs was confirmed by adherence, morphological changes, and surface expression of macrophage markers (**Figure 1**). From a morphological point of view, 87% of PMArest cells were adherent and an increase in size and granularity was observed, in line with previous studies (27). The differential combined positive staining for CD36, CD11b, CD14 and CD204 compared to THP-1 cells indicated that PMArest cells resemble macrophages also from a molecular point of view. This result is consistent with previous studies, in which CD36 in particular is often used as a marker of THP-1 differentiation into macrophages (22–24), and also reported to be expressed in alveolar macrophages isolated from COPD patients (31). CD11b has been formerly described as a macrophage specific marker in both human macrophages (32) and THP-1 derived macrophages (14, 15, 22). Our data confirmed the expression of CD11b at higher intensity in our PMArest population. CD14 is a membrane glycoprotein expressed on cells of the myelomonocyte lineage, mainly by macrophages. Increased CD14 expression in PMA-treated compared to untreated THP-1 cells has been previously described and partly explains the better response of macrophages to LPS and Aβ_1-42_ compared to monocytes (20). The lack of expression of CD14 in PMA48h group, along with heterogeneity in granularity and size detected by FACS analysis might indicate that the differentiation is still incomplete in this group. Noteworthy, the expression of CD14 was increased in PMArest, indicating a completed differentiation into MLC even at low concentration of PMA and validating the reported differentiation method. To check if the lack of CD14 in PMA48h group, compared to PMArest, may be due to the exposure length to PMA or to the time after the start of the PMA stimulus, we performed the FACS analysis for the expression of CD14 including a group of THP-1 cells treated with the same concentration of PMA for 72h. As reported in the **Supplementary Figure 1**, the percentage of cells expressing CD14 remains low (22%) also after 72h with PMA stimulus at 5ng/ml, while in the PMArest group about 70% of cells expressed the marker. This result suggests that not the timing after stimulus, but the resting period itself may play a role in the differentiation of THP-1 into MLCs.

NLRP3 inflammasome activation requires two signals, a priming signal that is typically provided by microbial components or endogenous cytokines and mediated by TLR4 activation and a second signal involving potassium ion efflux, lysosomal damage, particulate matter or reactive oxygen species generation. The priming signal may lead to the upregulation of NLRP3, which is thought to exist under resting conditions at too low concentrations to initiate inflammasome activation, and pro-IL-1β, which is not constitutively expressed in resting macrophages. The second signal causes the assembly of the inflammasome, a complex including NLRP3, ASC, and pro-caspase-1, with subsequent activation of caspase-1. Active caspase-1 cleaves pro-IL-1β and pro-IL-18 into their mature and biologically active forms (33). In THP-1 cells, PMA treatment has been shown to induce expression of IL-1β in a dose-dependent manner (20, 34). This aspect must be taken carefully into account when using the THP-1 derived macrophages as a model for the study of the inflammasome activation. In accordance with previous studies, our results confirm that even minimal amounts of PMA can induce the expression of pro-IL-1β in cell lysate and the secretion of IL-1β in the supernatant (**Figure 2**), thus PMA may provide not only for the priming but also for the second signal for the activation of the inflammasome.

Moreover, we report here for the first time the effect of PMA on the other mediators of the inflammasome, showing that the production of pro-IL-1β occurs alongside the overexpression of NLRP3 and decreased intracellular levels of pro-caspase-1 and ASC (**Figure 2**). Surprisingly, we were unable to detect any mature form of caspase-1, neither in the cell lysate, nor in the supernatant. Caspase-1 is normally present intracellularly in zymogen form, and after proteolytic cleavage, a tetrameric enzyme, (p20/p10)2, forms. The active subunits can be detected extracellularly, thus acting as a marker for inflammasome activation *in vitro* (35). Although the performed ELISA assay was validated by the analysis of caspase-1 in the CTRL+ sample, no appreciable level of the active enzyme was detected in any of the experimental groups, even in presence of released IL-1β in PMA48h group. One explanation may be that the activation of the NLRP3 inflammasome by PMA may be mediated by another caspase. Indeed, pro-IL-1β maturation and activation can be mediated by caspases other than caspase-1 or by other cell-type specific proteases, as recently reviewed by Pyrillou et al. (36). In particular, in dendritic cells and macrophages, cell stressors can promote caspase-8 dependent activation of NLRP3, and caspase-8 can provide proteolytic maturation of IL-1β in a caspase-1 independent manner (37). To address this hypothesis, we also analyzed the expression of caspase-8, however, no band for active caspase-8 in any experimental group was found (data not shown). Another explanation may be related to instability of the mature caspase-1. A previous study reported a very short half-life of the mature enzyme, with a full activity of about 9min. The authors related the rapid loss of activity to the instability of the quaternary structure of the active enzyme, resulting in loss of caspase-1 tetramers (38). On the other side, Shamaa and colleagues (39) distinguished between the activity of the enzyme in the cell extract, in which once activated, caspase-1 induced robust activity that was totally lost within an hour, and the activity of the released caspase-1, which is sustained even after immunodepletion of the enzyme. In our study, the time of collection, may have limited our chance to detect the enzyme through immunoblotting assay. An analysis of caspase-1 activity could help to fill the gap of understanding in the release of IL-1β in absence of its proteolytic enzyme.

To better understand the activation status of the inflammasome in PMA48h and PMArest, the formation of ASC specks was analyzed. ASC speck formation has been used as a readout for the inflammasome activation, since it serves as a signal amplification mechanism for inflammasome-mediated cytokine production, required for processing of IL-1β (1, 40). The speck formation is a quick process, so that upon activation the cytosolic levels of ASC drop down and all cytosolic ASC can be incorporated into a speck in <3min (41). In our study, western blot analysis showed a decrease of ASC levels in cell lysate of both PMA48h and PMArest groups compared to THP-1 cells, however ASC specks were not present in none of the two groups (**Figure 4**). Again, the observation timing may have limited our possibility to detect them. We then performed an extra analysis on THP-1 cells treated with 5ng/ml of PMA for 4h, 12h and 24h, but in none of the groups the ASC specks were detected (**Supplementary Figure 2**). This data, together with the missed detection of active caspase-1, may suggest that the PMA treatment at 5ng/ml provides the first signal for inflammasome activation, and so the substrates and changes in the intracellular levels of its mediators, but we are not able to prove if it also provides for its assembly in the functionally activated form. In conclusion, the presence of mature IL-1β in the PMA48h is open for further research, out of the scope of this work.

Of note, after 24h rest, no pro-IL-1β and only low amounts of released IL-1β were detected, suggesting that the rest allows for the removal of the inflammasome substrate. Since the release of IL-1β is considered by many authors as the primary outcome of the NLRP3 inflammasome activation, the 24h rest is mandatory to reduce the levels of IL-1β to the basal conditions and exclude biases in the interpretation of the results after exposure to subsequent stimuli. However, NLRP3 is still present, although at lower relative amount, and levels of pro-caspase-1 and ASC remain comparable to those in PMA48h group. We further evaluated the validity of the PMArest as a model for the study of the inflammasome, not only morphologically and molecularly similar to macrophage, but also able to respond to stimuli, by analyzing the ASC formation and the release of pro-inflammatory cytokines after canonical stimulation (LPS+NIG). The decrease of cytosolic ASC together with presence of ASC specks, in particular in PMArest cells treated with LPS followed by NIG, confirmed the ability of these cells to respond to stimuli by activation of the inflammasome. Moreover, the analysis of IL-1β demonstrated that, although the cytokine is not detected in untreated PMArest, the treatment with LPS alone or in combination with nigericin activates an inflammatory response leading to the production (pro-IL-1β) and the release (IL-1β) of the investigated cytokine (**Figure 3**). Again, the expression of CD14 may make PMArest cells more responsive to LPS compared to undifferentiated THP-1 (20, 42), so that also in the absence of the nigericin stimulus, the cells respond by complete activation of the inflammasome. This confirms our protocol as a valuable non-biased model for the study of the NLRP3 inflammasome, which can be used in the future to analyze other canonical stimuli, such as ATP, oxidized LDL or monosodium urate (MSU) crystals, where IL-1β can be used, but not exclusively, as readout parameter.

We conclude that treatment of THP-1 with 5ng/ml PMA followed by 24h rest period without PMA provides the differentiation into MLCs, from both a morphological and molecular point of view. A 24h rest period without PMA stimulus allows for a complete differentiation into a homogeneous population of MLCs and for the removal of IL-1β, but does not affect the responsiveness of the cells to subsequent stimuli, thus providing a non-biased *in vitro* model for the study of NLRP3 inflammasome.

## Data availability statement

The raw data supporting the conclusions of this article will be made available by the authors, without undue reservation.

## Author contributions

SG, EL-R, and MO contributed to conception and design of the study. SG performed the laboratory analysis. SG and EL-R contributed to the analysis interpretation. SG wrote the first draft of the manuscript. All authors contributed to manuscript revision, read, and approved the submitted version.
