# Supplementary material for: Resting time after phorbol 12-myristate 13-acetate in THP-1 derived macrophages provides a non-biased model for the study of NLRP3 inflammasome
Source: Front Immunol. 2022 Dec 22;13:958098. doi: 10.3389/fimmu.2022.958098 (PMC9817155; doi:10.3389/fimmu.2022.958098)
Supplement: Supplementary file 1 [file DataSheet_1.pdf]

Supplementary Material

Resting Time after Phorbol-12-Myristate-13-Acetate in THP-1 Derived Macrophages Provides a Non-biased Model for the Study of NLRP3 Inflammasome.

Sonia Giambelluca[1], Matthias Ochs[1,2] and Elena Lopez-Rodriguez[1#]

[1] Institute of Functional Anatomy, Charité - Univeristätsmedizin Berlin, Berlin, Germany

[2] German Center for Lung Research (DZL), Berlin, Germany

*** Correspondence:**
Elena Lopez-Rodriguez
elena.lopez-rodriguez@charite.de

1. Supplementary methods

All the methods used to obtain the supplementary results were already reported in the main document.

2. Supplementary results

2.1 Effect of PMA exposure length on CD14 expression

The differential expression of CD14 was assessed by FACS in THP-1 incubated without (THP-1) with 5 ng/ml PMA for 48h (PMA48h), with 5 ng/ml PMA for 48h plus 24h in fresh medium (PMArest), or with 5 ng/ml PMA for 72h (PMA72h). Supplementary figure 1 shows the histogram plots for CD14 expression assessed by comparison with matched isotype control, and the mean value of the percentage of total events expressing the single markers. THP-1 monocytes showed low surface expression of CD14 (16%). After treatment with PMA for 48h or 72h the percentage of cells expressing CD14 remained low (14% and 22%, respectively), while in the PMArest group about 70% of cells expressed the marker.

2.2 Immunofluorescence analysis of ASC specks

Oligomerization of ASC into specks, as a readout for inflammasome activation, was investigated in THP-1 cells treated with 5ng/ml of PMA for 4h (PMA4h), 12h (PMA12h) and 24h (PMA24h) to evaluate the effect of PMA over time on NLRP3 inflammasome. Representative results are shown in supplementary figure 2, where staining for ASC is represented in white and nuclear staining (Hoechst) in blue. Activation of the inflammasome is characterized by a change in ASC status from diffuse cytoplasmic form to a speck, visualized as a singular perinuclear structure. In any of the experimental groups, no speck structures were detected, and ASC showed a broad, weak cytosolic distribution.
To test the ability of the PMArest cells to respond to a canonical inflammasome activation stimulus, the immunofluorescence analysis of ASC specks was performed also in PMArest cells treated with LPS at 1 or 5µg/ml alone or followed by NIG at 5 or 10µM. Representative results

are shown in supplementary figure 3. After treatment with LPS alone at both concentrations tested, PMArest cells showed an amount of diffuse ASC decreased compared to untreated PMArest and few specks were detected. In PMArest treated with LPS followed by NIG, cytoplasmic ASC was barely detectable, while a high number of specks were visualized. No appreciable differences among the different concentration tested were observed.

3. Supplementary figures

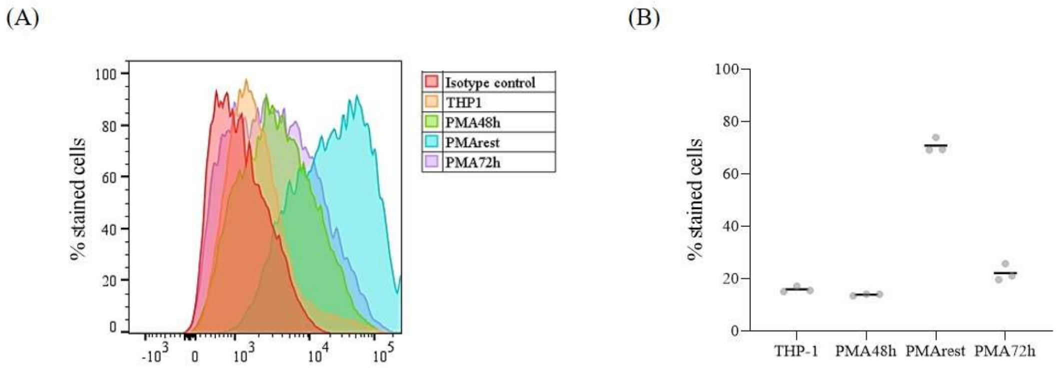

Supplementary Figure 1. Assessment of the differentiation into macrophage-like cells by flow cytometry. Representative FACS analysis of THP-1 incubated without (THP-1, yellow), with 5 ng/ml PMA for 48h (PMA48h, green), with 5 ng/ml PMA for 48h plus 24h in fresh medium (PMArest, blue), or with 5 ng/ml PMA for 72h (PMA72h, violet) cells stained using anti-CD14 conjugated antibody. For each marker, the expression was assessed by comparison with matched isotype control (red). (A) Histogram plots of marker expression (y: percentage of stained cells normalized by mode; x: fluorescence intensity). (B) Mean value of the percentage of total events expressing CD14. Data are shown as mean of three independent experiments in different plates with three different cell passages and freshly prepared stimuli (N=3).

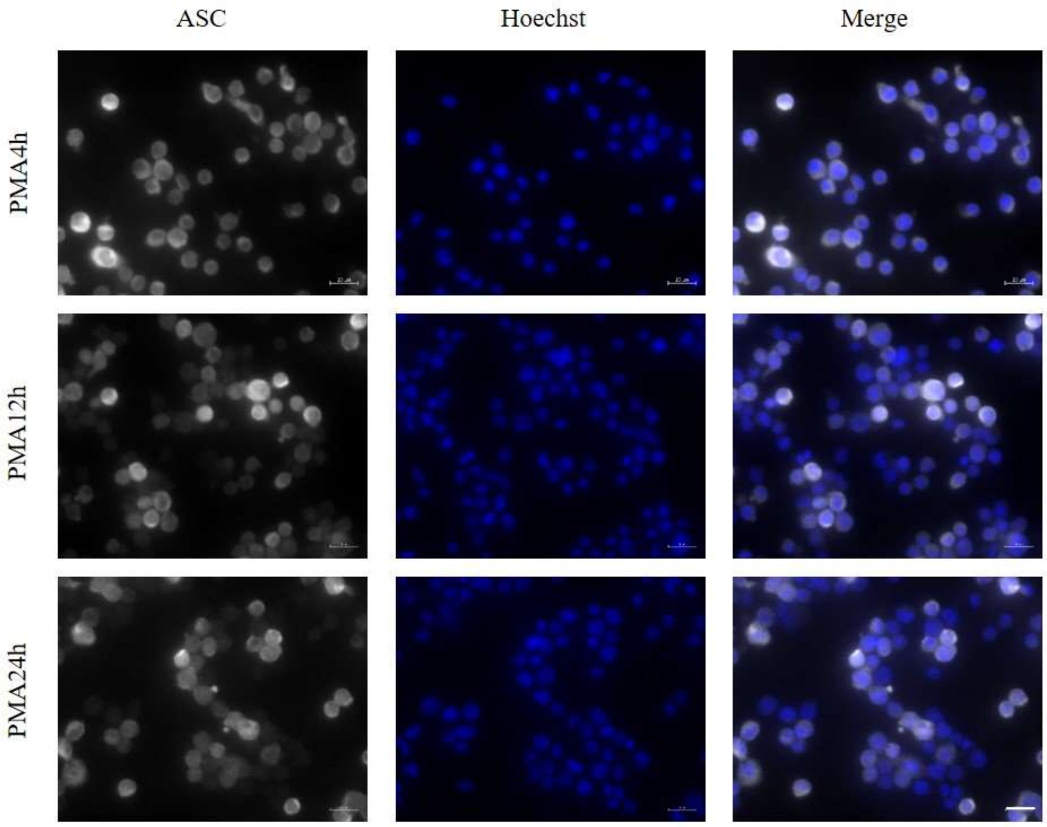

Supplementary Figure 2 Immunofluorescence analysis of ASC specks. Representative results of ASC speck formation in (from top to bottom) THP-1 cells incubated with 5 ng/ml PMA for 4h (PMA4h), 12h (PMA12h), and 24h (PMA24h). Cells were fixed and stained with anti-ASC antibody, followed by staining with PE-coniugated secondary antibody (colored in white). Nuclei were stained by incubation with Hoechst 34580 (false colored in blue). All the micrographs were taken at the same magnification and reported with the same scale (scale bar = 20µm).

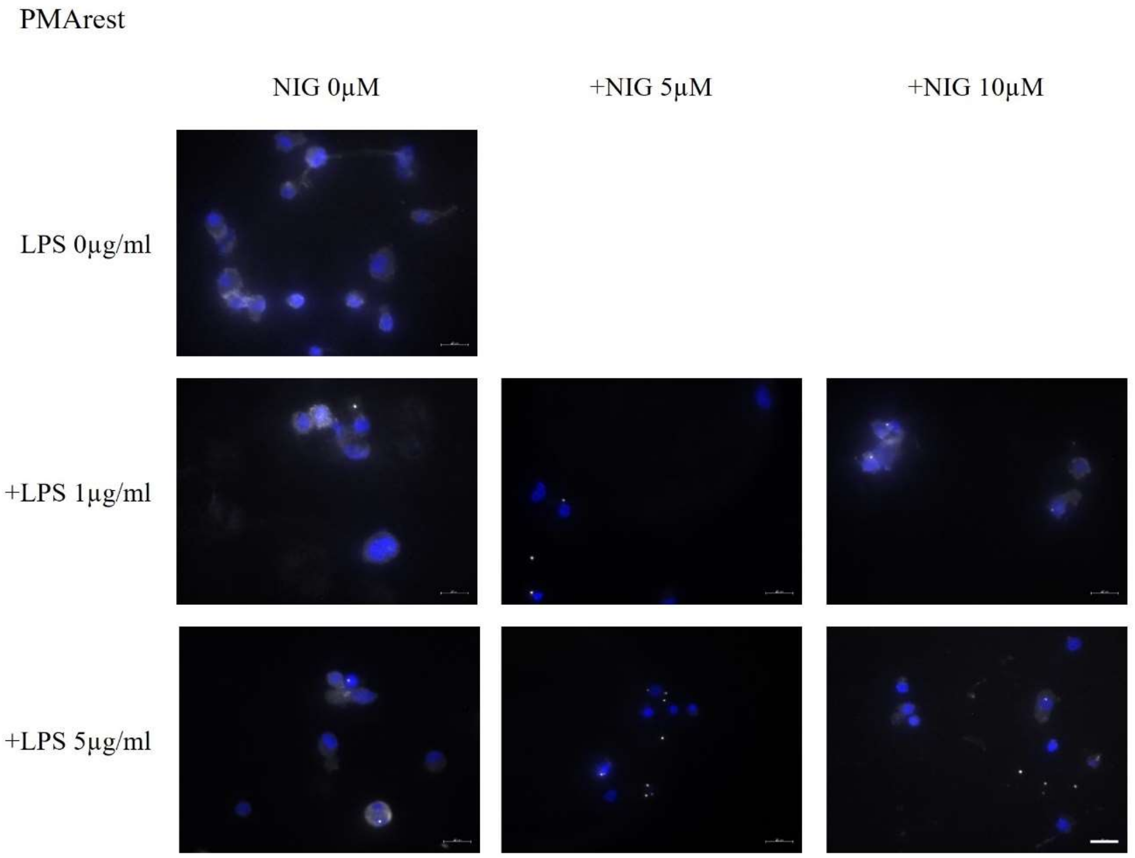

Supplementary Figure 3 Immunofluorescence analysis of ASC specks. Representative results of ASC speck formation in THP-1 cells incubated with 5 ng/ml PMA for 48h plus 24h in fresh medium (PMArest), treated with LPS at 1 or 5µg/ml alone (from top to bottom) or followed by nigericin (NIG) at 5 or 10µM (from left to right). Cells were fixed and stained with anti-ASC antibody, followed by staining with PE-coniugated secondary antibody (colored in white). Nuclei were stained by incubation with Hoechst 34580 (false colored in blue). All the micrographs were taken at the same magnification and reported with the same scale (scale bar = 20µm).
